# Supplementary material for: Influence of purple non-sulfur bacterial augmentation on soil nutrient dynamics and rice (Oryza sativa) growth in acidic saline-stressed environments
Source: PeerJ. 2024 May 17;12:e16943. doi: 10.7717/peerj.16943 (PMC11104354; doi:10.7717/peerj.16943)
Supplement: Supplemental Information 1 [file peerj-12-16943-s001.docx]

**Supplementary Table 1** Analysis of interactions between water salinity and purple nonsulfur bacteria on pH_water_.

| Factors | | Water salinity (A) (‰) | | | | Mean |
| --- | --- | --- | --- | --- | --- | --- |
|  |  | 0 | 2 | 3 | 4 |  |
| PNSB (B) (1.812 x 10^5^ CFU g^-1^ dry soil) | NAPNSB | 3.74±0.07^d^ | 3.75±0.04^d^ | 3.73±0.11^d^ | 3.81±0.02^bcd^ | 3.75±0.06^B^ |
|  | EPS18 | 3.81±0.04^bcd^ | 3.81±0.08^bcd^ | 4.04±0.12^ab^ | 4.05±0.12^ab^ | 3.92±0.09^A^ |
|  | EPS37 | 3.77±0.05^cd^ | 4.14±0.57^a^ | 4.05±0.15^ab^ | 3.86±0.05^bcd^ | 3.95±0.21^A^ |
|  | EPS54 | 3.70±0.07^d^ | 3.75±0.06^d^ | 4.02±0.17^abc^ | 3.90±0.05^a-d^ | 3.83±0.09^AB^ |
|  | MTPNSB | 3.87±0.08^bcd^ | 3.91±0.01^a-d^ | 3.85±0.07^bcd^ | 3.90±0.10^a-d^ | 3.88±0.06^A^ |
| Mean | | 3.77±0.06^B^ | 3.87±0.15^AB^ | 3.93±0.12^A^ | 3.90±0.07^A^ |  |
| F (A) | | | | | | * |
| F (B) | | | | | | * |
| F (A x B) | | | | | | * |
| CV (%) | | | | | | 3.88 |

*Note: NAPNSB: No application of purple non-sulfur bacteria*, *EPS18: application of EPS18 strain; EPS37: application of EPS37 strain; EPS54: application of EPS54 strain; MTPNSB: application of the mixture of strains EPS18, EPS37 and* *EPS54. The identical lowercase letters indicate insignificant differences between numbers in the same pool, while the identical uppercase letters indicate insignificant differences between numbers in the same column or row (P < 0.05).*

**Supplementary Table 2** Analysis of interactions between water salinity and purple nonsulfur bacteria on pH_KCl_.

| Factors | | Water salinity (A) (‰) | | | | Mean |
| --- | --- | --- | --- | --- | --- | --- |
|  |  | 0 | 2 | 3 | 4 |  |
| PNSB (B) (1.812 x 10^5^ CFU g^-1^ dry soil) | NAPNSB | 3.34±0.05^ab^ | 3.32±0.03^abc^ | 3.35±0.08^ab^ | 3.34±0.08^ab^ | 3.33±0.06 |
|  | EPS18 | 3.29±0.07^bc^ | 3.30±0.13^bc^ | 3.34±0.12^ab^ | 3.38±0.12^ab^ | 3.30±0.11 |
|  | EPS37 | 3.20±0.02^cd^ | 3.45±0.17^a^ | 3.32±0.02^abc^ | 3.25±0.04^bcd^ | 3.30±0.06 |
|  | EPS54 | 3.24±0.03^bcd^ | 3.15±0.09^d^ | 3.36±0.06^ab^ | 3.36±0.04^ab^ | 3.27±0.06 |
|  | MTPNSB | 3.30±0.01^bc^ | 3.28±0.07^bc^ | 3.27±0.04^bcd^ | 3.29±0.08^bc^ | 3.28±0.05 |
| Mean | | 3.27±0.04 | 3.29±0.10 | 3.32±0.06 | 3.32±0.07 |  |
| F (A) | | | | | | ns |
| F (B) | | | | | | ns |
| F (A x B) | | | | | | * |
| CV (%) | | | | | | 2.46 |

*Note: NAPNSB: No application of purple non-sulfur bacteria*, *EPS18: application of EPS18 strain; EPS37: application of EPS37 strain; EPS54: application of EPS54 strain; MTPNSB: application of the mixture of strains EPS18, EPS37 and* *EPS54. The identical lowercase letters indicate insignificant differences between numbers in the same pool, while the identical uppercase letters indicate insignificant differences between numbers in the same column or row (P < 0.05).*

**Supplementary Table 3** Analysis of interactions between water salinity and purple nonsulfur bacteria on Na content in soil.

| Factors | | Water salinity (A) (‰) | | | | Mean |
| --- | --- | --- | --- | --- | --- | --- |
|  |  | 0 | 2 | 3 | 4 |  |
| PNSB (B) (1.812 x 10^5^ CFU g^-1^ dry soil) | NAPNSB | 1.020±0.067^b^ | 1.158±0.024^a^ | 1.250±0.062^a^ | 1.150±0.083^a^ | 1.144±0.059^A^ |
|  | EPS18 | 0.948±0.053^bcd^ | 0.720±0.088^gh^ | 0.815±0.133^d-g^ | 0.868±0.010^c-f^ | 0.837±0.071^AB^ |
|  | EPS37 | 0.753±0.101^fgh^ | 0.843±0.051^d-g^ | 0.985±0.087^bc^ | 0.923±0.099^b-e^ | 0.875±0.084^B^ |
|  | EPS54 | 0.810±0.090^efg^ | 0.885±0.110^c-f^ | 0.908±0.125^b-e^ | 0.985±0.075^bc^ | 0.896±0.100^B^ |
|  | MTPNSB | 0.643±0.010^h^ | 0.800±0.051^efg^ | 0.885±0.096^c-f^ | 0.858±0.085^c-f^ | 0.796±0.060^C^ |
| Mean | | 0.835±0.064^B^ | 0.881±0.065^B^ | 0.968±0.101^A^ | 0.956±0.070^A^ |  |
| F (A) | | | | | | * |
| F (B) | | | | | | * |
| F (A x B) | | | | | | * |
| CV (%) | | | | | | 9.16 |

*Note: NAPNSB: No application of purple non-sulfur bacteria*, *EPS18: application of EPS18 strain; EPS37: application of EPS37 strain; EPS54: application of EPS54 strain; MTPNSB: application of the mixture of strains EPS18, EPS37 and* *EPS54. The identical lowercase letters indicate insignificant differences between numbers in the same pool, while the identical uppercase letters indicate insignificant differences between numbers in the same column or row (P < 0.05).*

**Supplementary Table 4** Analysis of interactions between water salinity and purple nonsulfur bacteria on dry biomass of stem and leaves.

| Factors | | Water salinity (A) (‰) | | | | Mean |
| --- | --- | --- | --- | --- | --- | --- |
|  |  | 0 | 2 | 3 | 4 |  |
| PNSB (B) (1.812 x 10^5^ CFU g^-1^ dry soil) | NAPNSB | 23.4±1.04^fgh^ | 23.4±1.13^fgh^ | 23.3±2.36^f-i^ | 19.9±2.04^j^ | 22.5±1.64^C^ |
|  | EPS18 | 26.4±0.48^cde^ | 23.4±0.73^fgh^ | 20.8±0.24^ij^ | 20.9±2.04^hij^ | 22.8±0.88^C^ |
|  | EPS37 | 24.0±0.57^efg^ | 24.4±1.43^efg^ | 23.3±1.02^f-i^ | 24.4±1.43^efg^ | 24.0±1.12^B^ |
|  | EPS54 | 29.1±1.73^b^ | 25.4±1.73^def^ | 22.0±2.48^g-j^ | 22.0±0.90^g-j^ | 24.6±1.71^B^ |
|  | MTPNSB | 31.9±1.89^a^ | 28.7±1.99^bc^ | 27.2±2.41^bcd^ | 24.2±1.10^efg^ | 28.0±1.85^A^ |
| Mean | | 27.0±1.14^A^ | 25.0±1.40^B^ | 23.3±1.71^C^ | 22.2±1.50^D^ |  |
| F (A) | | | | | | * |
| F (B) | | | | | | * |
| F (A x B) | | | | | | * |
| CV (%) | | | | | | 6.54 |

*Note: NAPNSB: No application of purple non-sulfur bacteria*, *EPS18: application of EPS18 strain; EPS37: application of EPS37 strain; EPS54: application of EPS54 strain; MTPNSB: application of the mixture of strains EPS18, EPS37 and* *EPS54. The identical lowercase letters indicate insignificant differences between numbers in the same pool, while the identical uppercase letters indicate insignificant differences between numbers in the same column or row (P < 0.05).*

**Supplementary Table 5** Analysis of interactions between water salinity and purple nonsulfur bacteria on dry biomass of grain.

| Factors | | Water salinity (A) (‰) | | | | Mean |
| --- | --- | --- | --- | --- | --- | --- |
|  |  | 0 | 2 | 3 | 4 |  |
| PNSB (B) (1.812 x 10^5^ CFU g^-1^ dry soil) | NAPNSB | 14.3±0.36^gh^ | 11.9±0.17^i^ | 11.4±0.92^ij^ | 10.5±0.34^j^ | 12.0±0.45^C^ |
|  | EPS18 | 16.6±0.59^de^ | 15.6±0.77^ef^ | 14.4±1.09^fgh^ | 14.7±0.53^fgh^ | 15.3±0.74^B^ |
|  | EPS37 | 18.2±0.65^ab^ | 16.5±0.34^de^ | 14.6±0.33^fgh^ | 13.6±0.85^h^ | 15.7±0.54^B^ |
|  | EPS54 | 17.8±1.22^bc^ | 16.5±0.41^de^ | 16.3±1.36^de^ | 14.9±1.28^fg^ | 16.3±1.07^A^ |
|  | MTPNSB | 19.1±0.77^a^ | 17.0±0.47^cd^ | 15.5±0.61^efg^ | 14.4±0.49^fgh^ | 16.5±0.59^A^ |
| Mean | | 17.2±0.72^A^ | 15.5±0.43^B^ | 14.4±0.86^C^ | 13.6±0.70^D^ |  |
| F (A) | | | | | | * |
| F (B) | | | | | | * |
| F (A x B) | | | | | | * |
| CV (%) | | | | | | 2.36 |

*Note: NAPNSB: No application of purple non-sulfur bacteria*, *EPS18: application of EPS18 strain; EPS37: application of EPS37 strain; EPS54: application of EPS54 strain; MTPNSB: application of the mixture of strains EPS18, EPS37 and* *EPS54. The identical lowercase letters indicate insignificant differences between numbers in the same pool, while the identical uppercase letters indicate insignificant differences between numbers in the same column or row (P < 0.05).*

**Supplementary Table 6** Analysis of interactions between water salinity and purple nonsulfur bacteria on total K uptake.

| Factors | | Water salinity (A) (‰) | | | | Mean |
| --- | --- | --- | --- | --- | --- | --- |
|  |  | 0 | 2 | 3 | 4 |  |
| PNSB (B) (1.812 x 10^5^ CFU g^-1^ dry soil) | NAPNSB | 322.2±18.1^i^ | 345.5±37.6^hi^ | 316.8±73.4^i^ | 303.9±31.5^i^ | 322.1±40.1^D^ |
|  | EPS18 | 543.0±17.4^c^ | 458.5±29.1^def^ | 444.6±9.2^d-g^ | 382.0±44.3^gh^ | 457.0±25.0^BC^ |
|  | EPS37 | 406.3±30.5^fg^ | 468.1±53.5^def^ | 423.1±21.4^d-g^ | 479.9±36.3^d^ | 444.3±35.4^C^ |
|  | EPS54 | 625.5±53.5^b^ | 471.1±58.9^de^ | 409.3±24.2^efg^ | 416.5±34.7^d-g^ | 480.6±42.8^B^ |
|  | MTPNSB | 693.0±56.0^a^ | 552.2±10.2^c^ | 562.4±45.7^c^ | 390.0±22.4^gh^ | 549.4±33.6^A^ |
| Mean | | 518.0±35.1^A^ | 459.1±37.8^B^ | 459.1±37.8^B^ | 394.5±33.8^D^ |  |
| F (A) | | | | | | * |
| F (B) | | | | | | * |
| F (A x B) | | | | | | * |
| CV (%) | | | | | | 8.54 |

*Note: NAPNSB: No application of purple non-sulfur bacteria*, *EPS18: application of EPS18 strain; EPS37: application of EPS37 strain; EPS54: application of EPS54 strain; MTPNSB: application of the mixture of strains EPS18, EPS37 and* *EPS54. The identical lowercase letters indicate insignificant differences between numbers in the same pool, while the identical uppercase letters indicate insignificant differences between numbers in the same column or row (P < 0.05).*

**Supplementary Table 7** Analysis of interactions between water salinity and purple nonsulfur bacteria on total Na uptake.

| Factors | | Water salinity (A) (‰) | | | | Mean |
| --- | --- | --- | --- | --- | --- | --- |
|  |  | 0 | 2 | 3 | 4 |  |
| PNSB (B) (1.812 x 10^5^ CFU g^-1^ dry soil) | NAPNSB | 131.5±8.7^c-f^ | 155.5±22.5^ab^ | 159.3±16.4^a^ | 142.5±19.9^a-d^ | 147.2±16.9^A^ |
|  | EPS18 | 107.1±9.1^fg^ | 99.9±15.7^g^ | 112.2±6.8^efg^ | 124.7±16.5^c-f^ | 111.0±12.0^C^ |
|  | EPS37 | 113.2±8.6^efg^ | 120.9±11.9^d-g^ | 122.1±9.2^d-g^ | 156.0±8.8^ab^ | 128.1±9.6^B^ |
|  | EPS54 | 130.2±15.4^c-f^ | 148.4±17.4^abc^ | 130.9±16.0^c-f^ | 133.2±7.8^b-e^ | 135.7±14.2^B^ |
|  | MTPNSB | 130.2±23.0^c-f^ | 120.7±20.6^d-g^ | 125.3±7.1^c-f^ | 127.9±10.0^c-f^ | 126.0±15.2^B^ |
| Mean | | 122.4±13.0^B^ | 129.1±17.6^AB^ | 130.0±11.1^AB^ | 136.8±12.6^A^ |  |
| F (A) | | | | | | * |
| F (B) | | | | | | * |
| F (A x B) | | | | | | * |
| CV (%) | | | | | | 11.4 |

*Note: NAPNSB: No application of purple non-sulfur bacteria*, *EPS18: application of EPS18 strain; EPS37: application of EPS37 strain; EPS54: application of EPS54 strain; MTPNSB: application of the mixture of strains EPS18, EPS37 and* *EPS54. The identical lowercase letters indicate insignificant differences between numbers in the same pool, while the identical uppercase letters indicate insignificant differences between numbers in the same column or row (P < 0.05).*


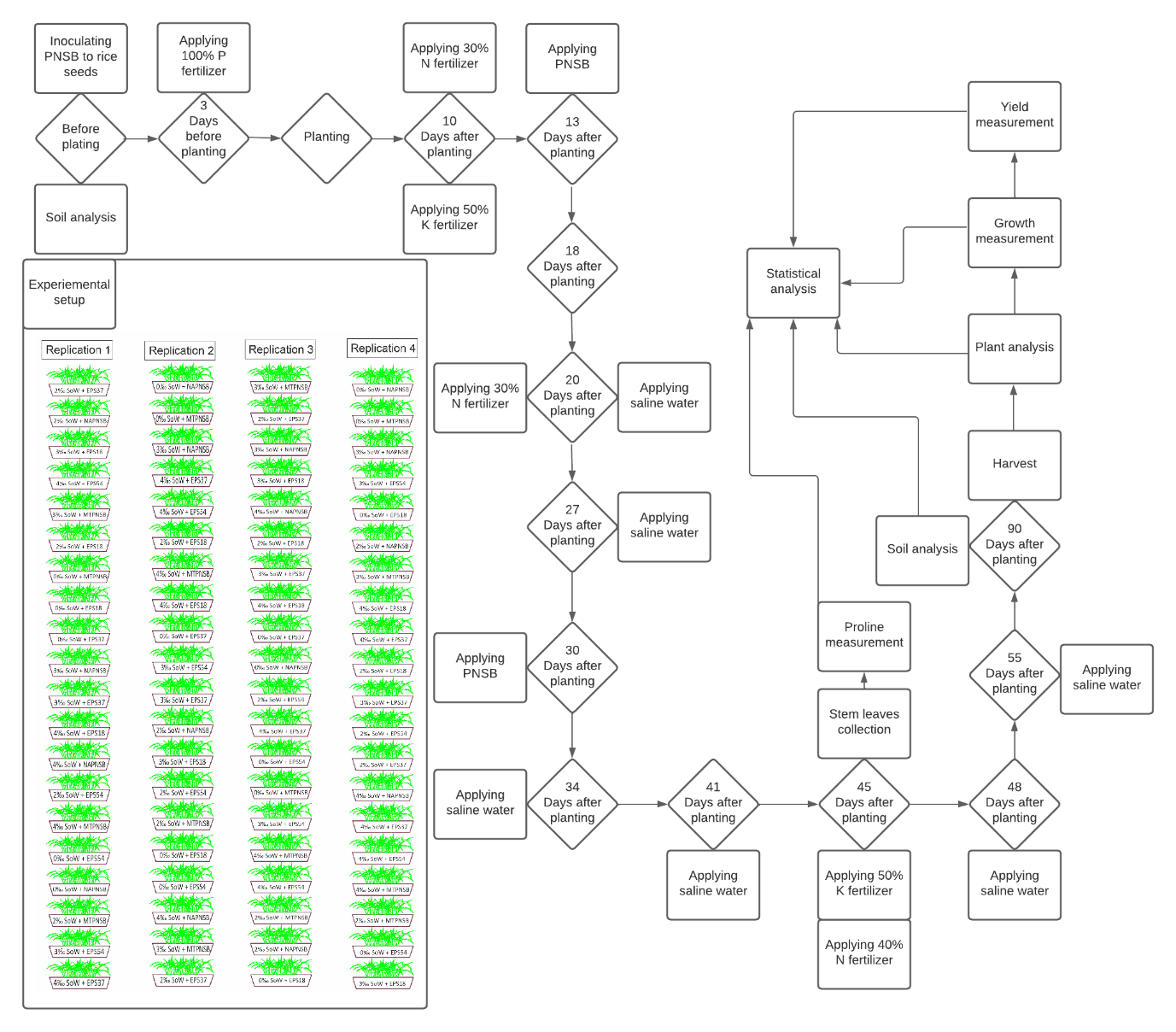


**Supplementary Figure 1** Research procedure.

**
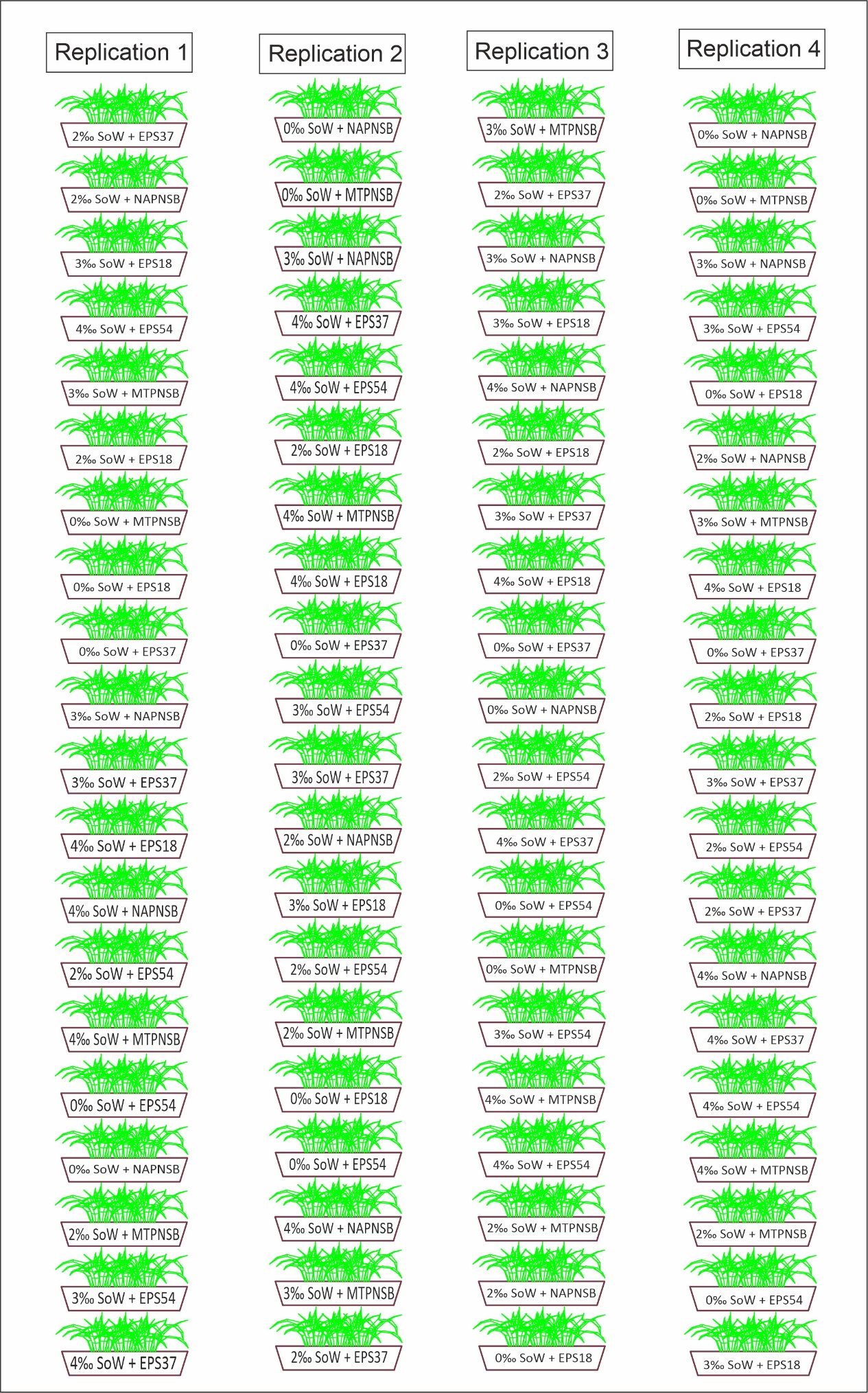
**

**Supplementary Figure 2** Diagram of experimental design.
