# Supplementary material for: Influence of purple non-sulfur bacterial augmentation on soil nutrient dynamics and rice (Oryza sativa) growth in acidic saline-stressed environments
Source: PeerJ. 2024 May 17;12:e16943. doi: 10.7717/peerj.16943 (PMC11104354; doi:10.7717/peerj.16943)
Supplement: Supplemental Information 2 [file peerj-12-16943-s002.docx]

The three sequences have been uploaded on the NCBI, and will be released on June 4^th^ 2026 under a GenBank submission: SUB13466767. The sequences were as follows:

>EPS18

AACGCGTGGGAACGTGCCCTTTGCTTCGGAATAGCCCCGGGAAACTGGGAGTAATACCGAATGTGCCCTTCGGGGGAAAGATTTATCGGCAAAGGATCGGCCCGCGTTGGATTAGGTAGTTGGTGGGGTAATGGCCTACCAAGCCGACGATCCATAGCTGGTTTGAGAGGATGATCAGCCACACTGGGACTGAGACACGGCCCAGACTCCTACGGGAGGCAGCAGTGGGGAATCTTAGACAATGGGCGCAAGCCTGATCTAGCCATGCCGCGTGATCGATGAAGGCCTTAGGGTTGTAAAGATCTTTCAGGTGGGAAGATAATGACGGTACCACCAGAAGAAGCCCCGGCTAACTCCGTGCCAGCAGCCGCGGTAATACGGAGGGGGCTAGCGTTATTCGGAATTACTGGGCGTAAAGCGCACGTAGGCGGATCGGAAAGTCAGAGGTGAAATCCCAGGGCTCAACCCTGGAACTGCCTTTGAAACTCCCGATCTTGAGGTCGAGAGAGGTGAGTGGAATTCCGAGTGTAGAGGTGAAATTCGTAGATATTCGGAGGAACACCAGTGGCGAAGGCGGCTCACTGGCTCGATACTGACGCTGAGGTGCGAAAGCGTGGGGAGCAAACAGGATTAGATACCCTGGTAGTCCACGCCGTAAACGATGAATGCCAGTCGTCGGGCAGCATGCTGTTCGGTGACACACCTAACGGATTAAGCATTCCGCCTGGGGAGTACGGCCGCAAGGTTAAAACTCAAAGGAATTGACGGGGGCCCGCACAAGCGGTGGAGCATGTGGTTTAATTCGAAGCAACGCGCAGAACCTTACCAACCCTTGACATGGCGATCGCGGTTCCAGAGATGGTTCCTTCAGTTCGGCTGGATCGCACACAGGTGCTGCATGGCTGTCGTCAGCTCGTGTCGTGAGATGTTCGGTTAAGTCCGGCAACGAGCGCAACCCACGTCCTTAGTTGCCAGCATTCAGTTGGGCACTCTAGGGAAACTGCCGGTGATAAGCCGGAAGAAAGTGTGGATGACGTCAAGTCCTCATGGCCCTTACGGGTTGGGCTACCACGTGCTACAATGGCAGTGAAAATGGGTTAA

>EPS37

ACGGGTGAGTAACGCGTGGGAACGTGCCCTTTGCTTCGGAATAGCCCCGGGAAACTGGGAGTAATACCGAATGTGCCCTTCGGGGGAAAGATTTATCGGCAAAGGATCGGCCCGCGTTGGATTAGGTAGTTGGTGGGGTAATGGCCTACCAAGCCGACGATCCATAGCTGGTTTGAGAGGATGATCAGCCACACTGGGACTGAGACACGGCCCAGACTCCTACGGGAGGCAGCAGTGGGGAATCTTAGACAATGGGCGCAAGCCTGATCTAGCCATGCCGCGTGATCGATGAAGGCCTTAGGGTTGTAAAGATCTTTCAGGTGGGAAGATAATGACGGTACCACCAGAAGAAGCCCCGGCTAACTCCGTGCCAGCAGCCGCGGTAATACGGAGGGGGCTAGCGTTATTCGGAATTACTGGGCGTAAAGCGCACGTAGGCGGATCGGAAAGTCAGAGGTGAAATCCCAGGGCTCAACCCTGGAACTGCCTTTGAAACTCCCGATCTTGAGGTCGAGAGAGGTGAGTGGAATTCCGAGTGTAGAGGTGAAATTCGTAGATATTCGGAGGAACACCAGTGGCGAAGGCGGCTCACTGGCTCGATACTGACGCTGAGGTGCGAAAGCGTGGGGAGCAAACAGGATTAGATACCCTGGTAGTCCACGCCGTAAACGATGAATGCCAGTCGTCGGGCAGCATGCTGTTCGGTGACACACCTAACGGATTAAGCATTCCGCCTGGGGAGTACGGCCGCAAGGTTAAAACTCAAAGGAATTGACGGGGGCCCGCACAAGCGGTGGAGCATGTGGTTTAATTCGAAGCAACGCGCAGAACCTTACCAACCCTTGACATGGCGATCGCGGTTCCAGAGATGGTTCCTTCAGTTCGGCTGGATCGCACACAGGTGCTGCATGGCTGTCGTCAGCTCGTGTCGTGAGATGTTCGGTTAAGTCCGGCAACGAGCGCAACCCACGTCCTTAGTTGCCAGCATTCAGTTGGGCACTCTAGGGAAACTGCCGGTGATAAGCCGGAAGAAGGGTGGATGACGTCAAGTCCTCATGGCCCTTACGGGTTGGGCTACCACGTGCTACATGGCAGTGAC

>EPS54

CGGCGGTACGGGTGAGTAACGCGTGGGAACGTGCCCTTTGCTTCGGAATAGCCCCGGGAAACTGGGAGTAATACCGAATGTGCCCTTTGGGGGAAAGATTTATCGGCAAAGGATCGGCCCGCGTTGGATTAGGTAGTTGGTGGGGTAATGGCCTACCAAGCCGACGATCCATAGCTGGTTTGAGAGGATGATCAGCCACACTGGGACTGAGACACGGCCCAGACTCCTACGGGAGGCAGCAGTGGGGAATCTTAGACAATGGGCGCAAGCCTGATCTAGCCATGCCGCGTGATCGATGAAGGCCTTAGGGTTGTAAAGATCTTTCAGGTGGGAAGATAATGACGGTACCACCAGAAGAAGCCCCGGCTAACTCCGTGCCAGCAGCCGCGGTAATACGGAGGGGGCTAGCGTTATTCGGAATTACTGGGCGTAAAGCGCACGTAGGCGGATCGGAAAGTCAGAGGTGAAATCCCAGGGCTCAACCCTGGAACTGCCTTTGAAACTCCCGATCTTGAGGTCGAGAGAGGTGAGTGGAATTCCGAGTGTAGAGGTGAAATTCGTAGATATTCGGAGGAACACCAGTGGCGAAGGCGGCTCACTGGCTCGATACTGACGCTGAGGTGCGAAAGCGTGGGGAGCAAACAGGATTAGATACCCTGGTAGTCCACGCCGTAAACGATGAATGCCAGTCGTCGGGCAGCATGCTGTTCGGTGACACACCTAACGGATTAAGCATTCCGCCTGGGGAGTACGGCCGCAAGGTTAAAACTCAAAGGAATTGACGGGGGCCCGCACAAGCGGTGGAGCATGTGGTTTAATTCGAAGCAACGCGCAGAACCTTACCAACCCTTGACATGGCGATCGCGGTTCCAGAGATGGTTCCTTCAGTTCGGCTGGATCGCACACAGGTGCTGCATGGCTGTCGTCAGCTCGTGTCGTGAGATGTTCGGTTAAGTCCGGCAACGAGCGCAACCCACGTCCTTAGTTGCCAGCATTCAGTTGGGCACTCTAGGGAAACTGCCGGTGATAAGCCGGAAGAAAGTGGGGATGACGTCAAGTCCTCATGGCCCTTACGGGTTGGGCTACCACCGTGCTACAATGGCAGTGACAATGGGTTAATCCCAAAAACTGTCTCCNTTTCGGATTGGGG
